# Associations Among Depression, Hemoglobin A1c Level, and Prognosis in Patients With Coronary Artery Disease: A Prospective Study

**DOI:** 10.3389/fpsyt.2022.815196

**Published:** 2022-06-16

**Authors:** Weiya Li, Han Yin, Quanjun Liu, Yilin Chen, Yanting Liang, Haofeng Zhou, Huan Ma, Qingshan Geng

**Affiliations:** ^1^Department of Cardiology, Guangdong Cardiovascular Institute, Guangdong Provincial People's Hospital, Guangdong Academy of Medical Sciences, Guangzhou, China; ^2^School of Medicine, South China University of Technology, Guangzhou, China; ^3^Department of Cardiac Rehabilitation, Guangdong Cardiovascular Institute, Guangdong Provincial People's Hospital, Guangdong Academy of Medical Sciences, Guangzhou, China

**Keywords:** depression, coronary artery disease, HbA1c, prognosis, MACE

## Abstract

**Background:**

Depression is ubiquitous in patients with coronary artery disease (CAD). The relationship between depression and hemoglobin A1c (HbA1c) is controversial. The combined effect of high HbA1c and depression on prognosis is unclear, especially in non-diabetic CAD patients. We sought to explore these associations.

**Methods:**

558 CAD patients were included in this prospective study. Patients were grouped by HbA1c levels and the status of clinical depression. The average follow-up period was about 2.2 years, and Cox proportional hazards models were used to compare the differences of prognosis in all the groups.

**Results:**

Clinical depression had no associations with HbA1c in all CAD patients (P for Pearson correlation = 0.74). In the all four groups, compared to group 1 (patients without clinical depression and low HbA1c), group 3 (without clinical depression and high HbA1c) had a higher risk of MACE (adjusted hazard ratio [aHR], 1.97; 95% confidence interval [CI], 1.2–3.25) and composite events (aHR, 1.67; 95% CI, 1.09–2.053). Group 4 (patients with clinical depression and high HbA1c) had higher HRs for MACE (aHR, 2.9; 95%CI, 1.32–6.38) and composite events (aHR, 2.12; 95% CI, 1.06–4.25). In CAD patients without diabetes, patients with clinical depression and high HbA1c had a higher risk of MACE (HR, 2.71; 95% CI, 1.02–7.19), non-cardiac readmission (HR,3.48; 95% CI, 1.26–9.57) and composite events (HR,2.44; 95% CI, 1.08–5.53) than those with no clinical depression and low HbA1c. In patients with comorbidities of depression and diabetes, patients with depression and high HbA1c more likely to experienced non-cardiac readmissions (HR, 4.49; 95% CI, 1.31–15.38) than patients with no depression and low HbA1c only. In all the above analysis, p-values for interaction between clinical depression and HbA1c were not statistically significant.

**Conclusions:**

The presence of both depression and high HbA1c lead to a worse prognosis in CAD patients than one risk factor alone, no matter with or without the comorbidity of diabetes in these CAD patients. For patients with CAD and depression, lower HbA1c may be required.

## Introduction

Coronary artery disease (CAD) is one of the major forms of cardiovascular diseases. It is well known that CAD is prevalent worldwide and has become a primary health concern in the general population over the past several decades (1). Depression is a prevalent mood disorder in patients with CAD. And numerous studies suggest that depression is highly correlated with the onset, development of CAD and means more cardiovascular morbidities and mortalities in CAD individuals (2–8). The prevalence of the comorbidity of depression and CAD in hospitalized patients have even reached 51% in China (9). In recent years, depression and glucose metabolism in CAD patients have received increasing and widespread attention (10–15).

HbA1c, a biomarker reflecting blood glucose status over the preceding 2–3 months, is considered the vital standard for predicting microvascular disease and atherosclerosis (16, 17). Previous studies have also shown that elevated HbA1c levels probably lead to dyslipidemia, hypercoagulability, and system inflammatory response which are well-established predictors of future mortality and cardiac events (18–22). And a positive association between elevated baseline HbA1c level and poor outcomes in CAD patients with and without diabetes has been reported (23–26).

HbA1c, a biomarker reflecting blood glucose status over the preceding 2–3 months, is considered the vital standard for predicting microvascular disease and atherosclerosis (8, 9). HbA1c is believed to be a risk factor of all-cause mortality and cardiac events (10–12). Previous studies have shown that elevated HbA1c levels probably lead to dyslipidemia, hypercoagulability, and system inflammatory response which are well-established predictors of future mortality (13, 14). And elevated baseline HbA1c level has a positive association with poor outcomes in CAD patients with and without diabetes (15–18).

Numerous studies have shown a significant correlation between depression and HbA1c (27–30). While several researchers found no correlations between depression and HbA1c (31, 32). Previous studies have focused on the association of HbA1c as well as depression and some debates existed, and only separate prognostic effect of depression and HbA1c was explored (20, 27, 28, 33). No investigators have yet explored the co-effects of depression and HbA1c levels to poor prognosis, especially about non-cardiac readmissions, in CAD patients. Our study aims to answer these questions.

## Methods

### Study Design and Participants

This is a prospective study conducted in Guangdong Provincial People's Hospital, Guangzhou, China to investigate the association between depression, HbA1c, and poor prognosis in CAD patients. 558 patients with CAD (at least one epicardial coronary artery stenosis ≥50% by coronary angiography surgery) from October 2017 to January 2018 were included in the analysis. Our psycho-cardiologist explained the psychiatric scale to all the patient one day before the coronary angiography and helped those with impaired vision or poor reading ability to complete the scales. We excluded those who did not complete the Patient Health Questionnaire-9. Those with missing HbA1c data and other cardiac severe comorbidities were also excluded. The study was approved by the Medical Ethics Committee of Guangdong Provincial People's Hospital. Written informed consent was obtained from all participants. Details about recruitment and excluded methods have been declared in our previous article (11).

### Follow-Up

All patients were followed up yearly by telephone or in-person for a total of 29 months. The average follow-up time was 26.3 ± 0.9 months. Major Adverse Cardiovascular Events (MACE) was defined as cardiac death, unplanned secondary revascularization, cardiac rehospitalization, non-fatal myocardial infarction and stroke. The composite endpoint included all-causes mortality, readmission for any reason and all kinds of MACEs. Readmissions were divided into cardiac and non-cardiac readmissions.

### Questionnaire and Other Data

Patient Health Questionnaire-9 (PHQ-9) was widely used to assess depression and the Chinese version of PHQ-9 has been validated in Chinese cardiac patients (34). The respondents were asked 9 questions about specific symptoms, assigning values of 0 to 3 points (0-not at all, 1-several days, 2-more than half of the days, 3-nearly every day) with the higher score on each item representing more frequently being bothered by the symptom in the last 2 weeks. PHQ-9 has been demonstrated to be a reliable predictor of depression and getting a score ≥ 5 means having depression (35). The cut-off score of 10 or more was used to determine clinical depression, which was showed to have 89% sensitivity and 89% specificity (6).

HbA1c was used to evaluate each participant's condition of diabetes control. The Cockcroft-Gault formula was used to calculate the creatinine clearance (CCR) through the serum creatinine tested at admission (36). The socio-demographic characteristics included age, gender, education, economic situation, smoking status, marital status as well as clinical data like diabetes medication, family medical history, hypertension, and relevant laboratory indicators were collected.

### Groupings

The HbA1c levels were assessed by comparing with the median value of the HbA1c value in the corresponding crowd. All of the 558 patients were divided into four groups according to the depression and HbA1c level. Patients without clinical depression and low HbA1c were defined as group 1, patients with clinical depression and low HbA1c were defined as group 2. Group 3 included participates without clinical depression and high HbA1c, and those with clinical depression and high HbA1c were defined as group 4.

Patients without clinical depression and low HbA1c were re-defined as group1' and group 1” in the non-diabetes and diabetes subgroup analysis, respectively. Similarly, patients with clinical depression and high HbA1c were divided into group3' and group3”. Others were divided into group2' and group2”.

### Statistical Analyses

The data was described in the form of mean ± SD, median (interquartile range), or number and percentage when appropriate. One-way analyses of variance and Kruskal-Wallis rank-sum tests were used to analyze continuous variables. When analyzing categorical data, we used the Pearson chi-squared test.

Pearson correlation was used to examine the association between clinical depression and HbA1c. Cox proportional hazards regression models were used to evaluate the influence of the groups with depression and HbA1c level on MACE and composite events in CAD patients. We compared the baseline data first and put the variables with a baseline difference level of <0.05 in the univariate Cox regression analysis. Variables with a significance level of 0.05 in the univariate Cox regression analysis [MACE: p for High-Density Lipoprotein Cholesterol (HDLC) = 0.044; Composite endpoint: p for CCR = 0.042; p for sex = 0.035; Non-cardiac readmissions: p for sex = 0.005; p for taking furosemide = 0.016; p for CCR < 0.001] were included in the final multivariate Cox regression. Other variables which were considered to be most closely associated to endpoint events, such as age, severity of coronary artery stenosis and diabetes were also regarded as necessary adjustment variables in the multivariate Cox regression. We adjusted sex, age, severity of coronary artery stenosis, diabetes and HDLC in the final multivariate MACE Cox regression. We adjusted sex, age, severity of coronary artery stenosis, diabetes and CCR in the final multivariate composite endpoint Cox regression. And in the final multivariate non-cardiac readmissions Cox regression, sex, age, severity of coronary artery stenosis, diabetes, taking furosemide, and CCR were adjusted.

P for interaction of depression and HbA1c for the outcomes were examined in all the Cox regressions. The level of significance was set at *p* < 0.05. Data was analyzed using SPSS version 25.

## Results

### Baseline Characteristics

The baseline of clinical characteristics of 558 CAD patients included in this analysis was shown in Table 1. The mean age of the participants was 63.7 ± 10.1 years, 430 (77.1%) were male. 440 (78.9%) and 118 (21.1%) were diagnosed with angina pectoris, myocardial infarction, respectively. A total of 340 (60.9%) had hypertension, and 195 (34.9%) were diagnosed with diabetes. 210 (37.6%) individuals had mild to severe depression. 62 participates (11.1%) were diagnosed with clinical depression. There were 290 patients with the HbA1c values below the median level (HbA1c ≤ 6.2%). All patients were divided into four groups: group 1 (no clinical depression and HbA1c ≤ 6.2, *n* = 258), group 2 (with clinical depression and HbA1c ≤ 6.2, *n* = 32), group 3 (no clinical depression and HbA1c>6.2, *n* = 238), group 4 (with clinical depression and HbA1c>6.2, *n* = 30). No association was found between HbA1c and clinical depression (P for Pearson correlation was 0.74).

**Table 1:** Baseline characteristic of all CAD patients grouped by clinical depression and HbA1c level.

**Varites**	**Overall**	**Groupings**				
	**(*n* = 558)**	**Group1 (*n* = 258)**	**Group2 (*n* = 32)**	**Group3 (*n* = 238)**	**Group4 (*n* = 30)**	***p***
**Demographic**						
Age, year	63.7 ± 10.1	63.6 ± 10.3	64.1 ± 9.8	63.8 ± 1.0	64.4 ± 10.9	0.97
Male,n (%)	430 (77.1)	212 (82.2)	21 (65.6)	175 (73.5)	22 (73.3)	0.044
BMI,kg/m^2^	24.5 ± 3.1	24.3 ± 2.9	23.8 ± 3.6	24.7 ± 3.0	24.8 ± 3.9	0.21
Education,n (<6 years/6-9 years/10-12 years/>12 years)	153/154/118/124	64/76/56/58	13/7/3/7	63/65/52/56	13/6/7/3	0.24
Marriage,n						
Married/Divorced or Widowed or Single	525/33	248/10	27/5	223/15	27/3.	0.078
**Medication**						
β-blockers,n (%)	486 (87.1)	218 (84.5)	27 (84.4)	214 (89.9)	27 (90.0)	0.29
CCB,n (%)	130 (23.3)	61 (23.6)	9 (28.1)	52 (21.8)	8 (26.7)	0.82
Taking furosemide, n (%)	67 (12.0)	17 (6.6)	4 (12.5)	39 (16.4)	7 (23.3)	0.002
**Medical history**						
Diabetes mellitus,n (%)	195 (34.9)	16 (6.2)	1 (3.1)	155 (65.1)	23 (76.7)	<0.001
Hypertension,n (%)	340 (60.9)	146 (56.6)	20 (62.5)	152 (63.9)	22 (73.3)	0.18
Family history of Diabetes, n (%)	91 (16.3)	23 (8.9)	4 (12.5)	58 (24.4)	6 (20.0)	<0.001
Family history of CAD,n (%)	97 (17.4)	37 (14.4)	4 (12.5)	47 (19.7)	9 (30.0)	0.092
Family history of Hypertesion, n (%)	173 (31.0)	75 (29.1)	10 (31.3)	76 (31.9)	12 (40.0)	0.64
**Laboratory and technical test**						
Severity of coronary artery stenosis,n (1/2/3)	108/112/338	64/54/140	8/10/14	35/43/160	1/5/24.	0.002
CCR,ml/min/1.73 m2	63.1 (50.9,77.4)	64.1 (53.5,78.9)	52.5 (45.1,69.0)	63.3 (48.6,77.8)	56.4 (43.5,79.8)	0.012
LDLC,mmol/L	2.9 ± 0.9	2.9 ± 0.9	2.7 ± 0.7	2.9 ± 0.9	3.1 ± 1.2	0.54
TC,mmol/L	4.2 (3.5,5.0)	4.2 (3.5,5.0)	4.2 (3.3,4.9)	4.3 (3.5,5.0)	4.0 (3.5,5.9)	0.94
HScrp,mg/L	2.5 (0.9,8.1)	1.8 (0.7,5.9)	2.9 (0.8,10.8)	2.8 (1.1,10.2)	5.9 (1.0,12.5)	0.017
CKMB,U/L	10.4 (8.3,13.1)	10.3 (8.0,12.7)	9.9 (7.9,11.9)	10.6 (8.6,14.1)	10.6 (8.3,13.6)	0.088
HDLC,mmol/L	0.9 (0.8,1.1)	1.0 (0.8,1.1)	1.0 (0.8,1.2)	0.9 (0.8,1.1)	0.9 (0.7,1.1)	0.028

*BMI, body mass index; CCB, calcium channel blockers; LVEF, left ventricular ejection fractions; CCR, creatinine clearance rate; LDL-C, low-density lipoprotein cholesterol; TC, total cholesterol; HDLC, high density liptein cholesterol; CKMB, creatine kinase isoenzymes*.

*Group1: patients without clinical depression and low HbA1c; Group2: patients with clinical depression and low HbA1c; Group3: patients without clinical depression and high HbA1c; Group4: patients with clinical depression and high HbA1c*.

### Main Outcomes

**Figure 1:**
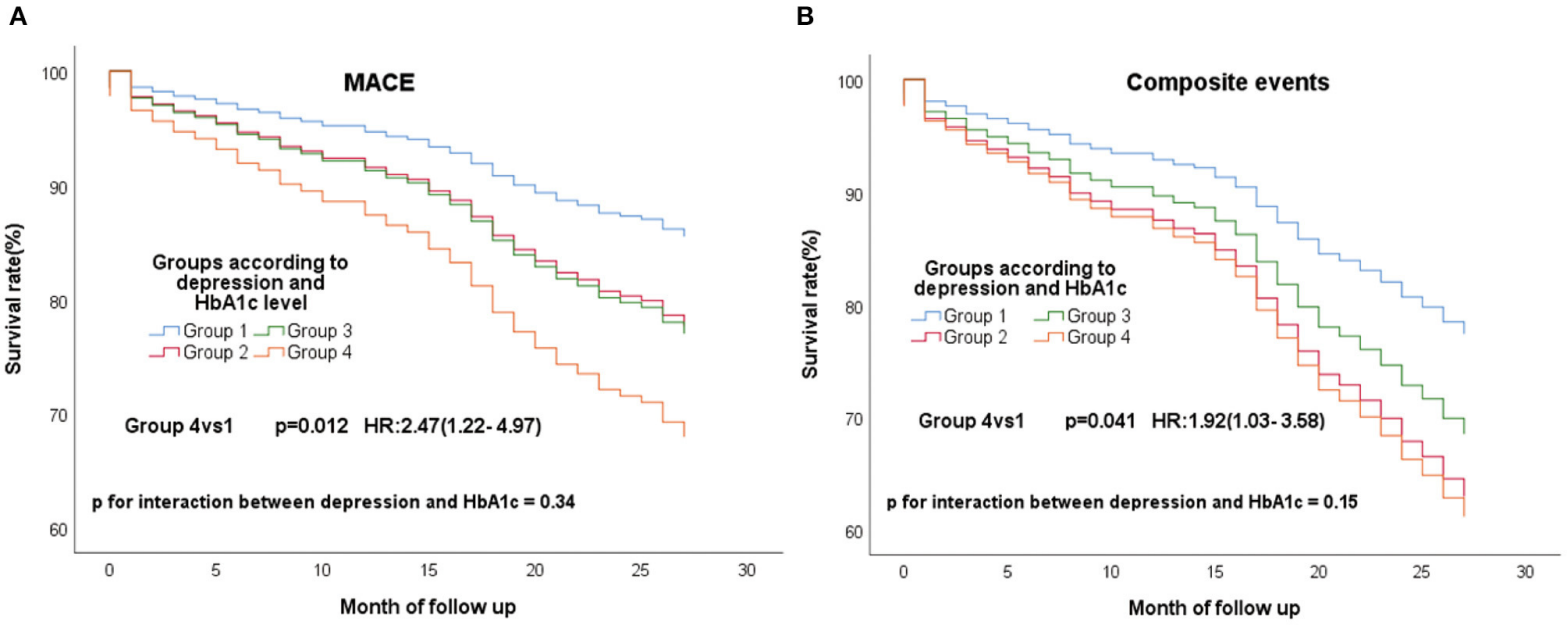
Cox regression curves for MACE **(A)** and composite endpoint **(B)** by the four groups of all patients. (Group1: patients without clinical depression and low HbA1c; Group2: patients with clinical depression and low HbA1c; Group3: patients without clinical depression and high HbA1c; Group4: patients with clinical depression and high HbA1c).

**Figure 2:**
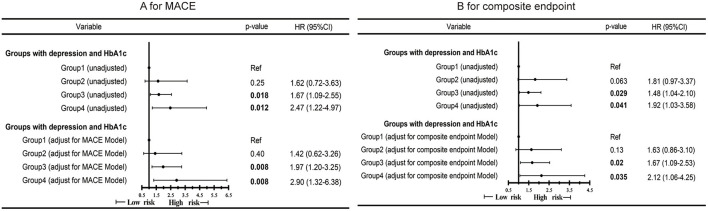
Cox proportional hazard ratios for MACE **(A)** and composite endpoint **(B)** by the four groups of all CAD patients in different models. MACE Model: Adjusted for sex, age, severity of coronary artery stenosis, diabetes and HDLC. Composite endpoint Model: Adjusted for sex, age, severity of coronary artery stenosis, diabetes and CCR.

During a mean follow-up of approximately 26.3 months, 151 (27.1%) and 105 (18.8%) experienced composite endpoint event and MACE, respectively. 66 (11.8%) experienced a non-cardiac readmission. In the Cox analysis, we found that group 4 had a higher risk of MACE (Figure 1A; *P* = 0.012; HR, 2.47; 95%CI, 1.22–4.97) and the composite events (Figure 1B; *P* = 0.041; HR, 1.92; 95% CI, 1.03–358) than group 1. P for interaction between depression and HbA1c for MACE was 0.34. P for interaction between depression and HbA1c for composite events was 0.15. After multi-variable adjustment, we found group 4 remained at higher risk for MACE (Figure 2A; *P* = 0.008; HR, 2.90; 95%CI, 1.32–6.38) and the composite events (Figure 2B; *P* = 0.035; HR, 2.12; 95% CI, 1.06–4.25) than group 1. Moreover, group 3 had a higher risk of MACE (Figure 2A; *P* = 0.008; HR, 1.97; 95% CI, 1.20–3.25) and composite events (Figure 2B; *P* = 0.02; HR, 1.67; 95% CI, 1.09–2.53) than group 1 in the multivariate adjusted model, too. While no significant differences were found in all the four groups for non-cardiac readmission (Supplementary Figure 1).

### Subgroup Analyses

We performed the subgroup analyses to explore whether similar findings existed in CAD patients with or without diabetes.

**Figure 3:**
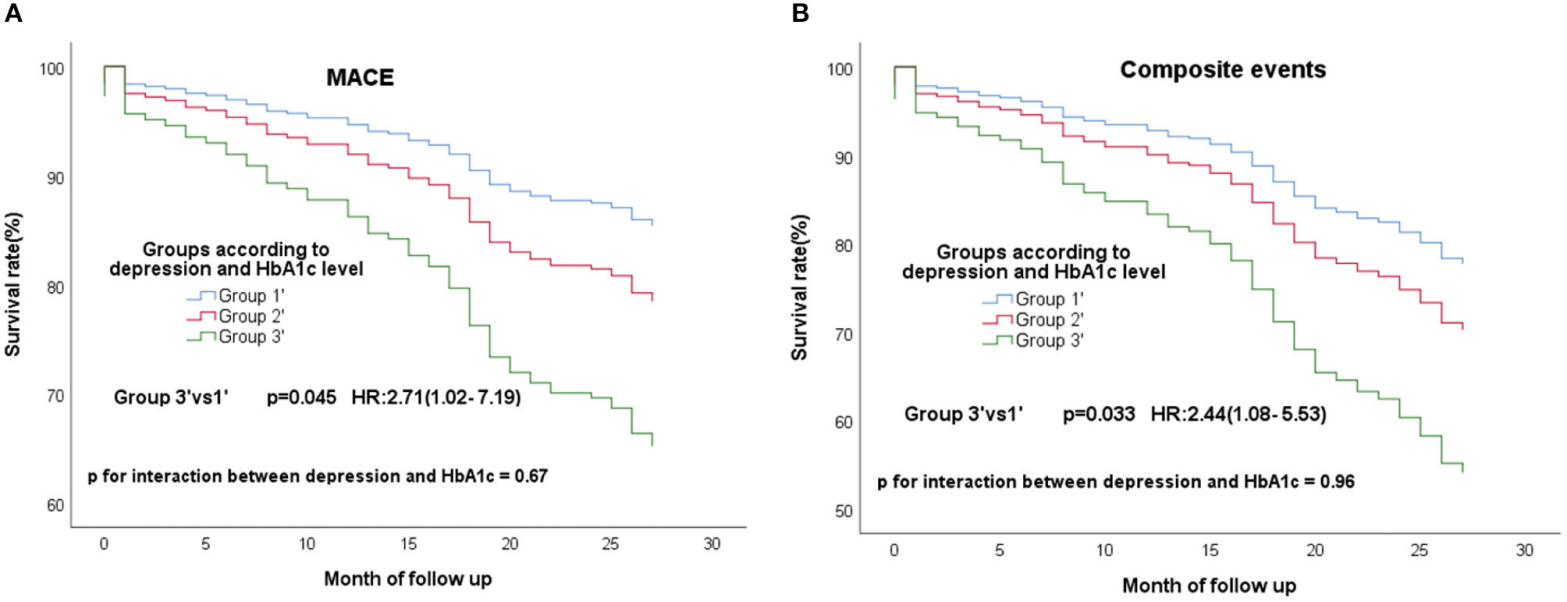
Cox regression curves for MACE **(A)** and composite endpoint **(B)** by the four groups of CAD patients without diabetes. (Group1': patients without clinical depression and low HbA1c; Group2': patients with clinical depression and low HbA1c, patients without clinical depression and high HbA1c; Group3': patients with clinical depression and high HbA1c).

**Figure 4:**
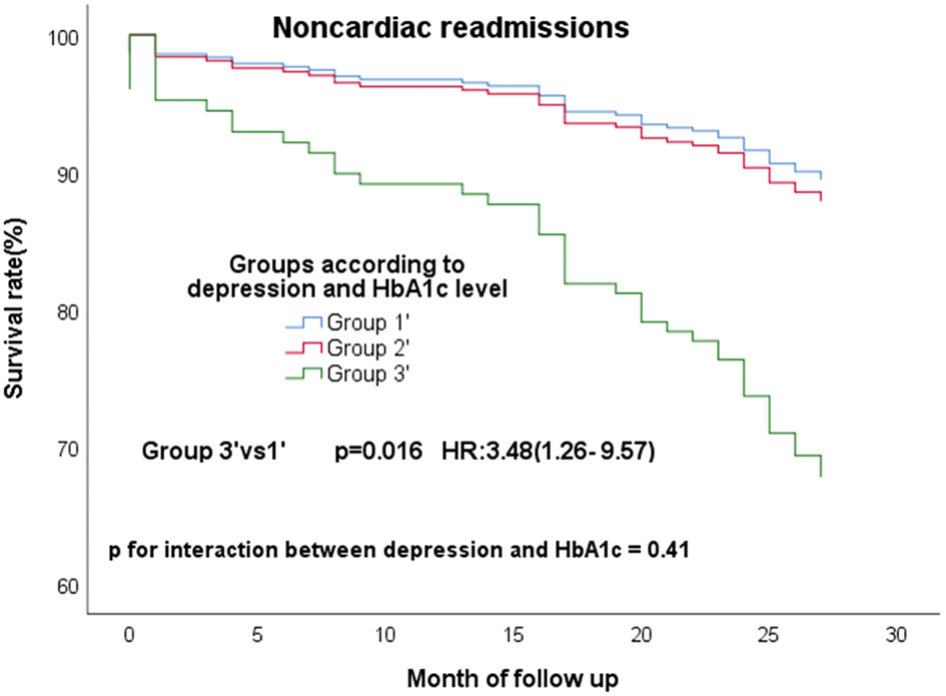
Cox regression curves for noncardiac readmission by the four groups of CAD patients without diabetes. (Group1': patients without clinical depression and low HbA1c; Group2': patients with clinical depression and low HbA1c, patients without clinical depression and high HbA1c; Group3': patients with clinical depression and high HbA1c).

In the all 363 CAD patients without diabetes, 67 (18.5%) experienced MACE and 96 (26.4%) experienced composite events. We re-divided these 363 people into high (≥5.9%) and low (<5.9%) HbA1c groups according to the median of HbA1c. These non-diabetic patients were re-grouped into three groups according to the depression and HbA1c level−149 patients without clinical depression and low HbA1c (group1'), 15 patients with clinical depression and high HbA1c (group3'), the residual other patients were defined as group2'. The same conclusion remained, group3' still had a high risk of composite events (Figure 3A; *P* = 0.045; HR, 2.71; 95% CI, 1.02–7.19) and MACE (Figure 3B; *P* = 0.033; HR, 2.44; 95% CI, 1.08–5.53) than group 1'. What's more, group3' had a higher risk of non-cardiac readmission, too (Figure 4; *P* = 0.016; HR, 3.48; 95% CI, 1.26–9.57). P for interactions between depression and HbA1c in all the above Cox regression models were < 0.05 (MACE: 0.67; Composite events: 0.96; Non-cardiac readmission: 0.41).

**Figure 5:**
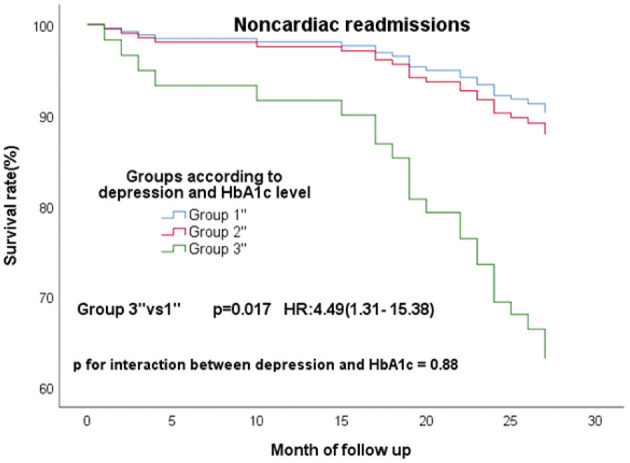
Cox regression curves for noncardiac readmission by the four groups of CAD patients with diabetes. (Group1”: patients without clinical depression and low HbA1c; Group2”: patients with clinical depression and low HbA1c, patients without clinical depression and high HbA1c; Group3”: patients with clinical depression and high HbA1c).

There were 195 patients with the comorbidity of CAD and diabetes. 38 (19.5%), 55 (28.2%) and 23 (11.8%) experienced MACE, composite events and non-cardiac readmissions, respectively. In these 195 patients, low and high HbA1c groups were defined as HbA1c less than the 50th percentile (7.7%) and HbA1c ≥7.7%, respectively. 77 patients without clinical depression and low HbA1c was defined as group1”. 11 patients with clinical depression and high HbA1c were divided into group3”, and others were included in the group2”. Something different was found, group3” had a high risk of non-cardiac readmissions (Figure 5; *P* = 0.017; HR, 4.49; 95% CI, 1.31–15.38) than group1” only. P for interaction between depression and HbA1c for non-cardiac readmission was 0.88. While no significant differences were found in the three groups for MACE and composite events (Supplementary Figure 2).

Due to the low number of endpoint events, no multifactor Cox regression analysis was carried out in the above two subgroup analyses.

## Discussion

To our knowledge, this is the first study to explore the relationship between HbA1c levels and depression on MACE events and noncardiac rehospitalization in CAD patients.

In our study, no correlation was found between clinical depression and HbA1c. This is consistent with the findings of a previous study by Josephine Akpalu et al. (32). As for all the CAD populations, higher HbA1c led to an increased probability of MACE and composite endpoint events **(HR for MACE and composite events: 1.97, 1.67)**. This was consistent with the conclusions of many previous studies (23, 26, 37, 38). What's more, when combined with clinical depression, the prognosis got even worse **(HR for MACE and composite events: 2.9, 2.12)**. However, clinical depression alone did not significantly worse the prognosis in the low HbA1c level groups (group2 vs group1), which warned us to pay more attention to CAD patients with the comorbidity of clinical depression and poor glycemic control (high HbA1c level).

Elevated inflammatory activity was proved as a potential mechanism causing adverse cardiovascular events of depression (8). A higher level of HbA1c is clearly associated with higher inflammatory biomarkers such as high-sensitivity C-reactive protein, leukocyte counts, fibrinogen, D-dimer and so on. Individually or in combination, these inflammatory factors associated with hyperglycemia had a direct role on the progression of atherosclerotic artery disease and adverse cardiovascular events (18, 26, 39–43). The dual inflammatory effect of both depression and high HbA1c levels may lead to the prognosis significantly worse.

Most of previous studies have only explored the separate effects of depression and HbA1c levels on the prognosis. Moreover, they did not focus on non-cardiac readmissions. Our subgroup analysis illustrated that in CAD patients without diabetes, clinical depression and high HbA1c significantly associated with MACE, non-cardiac readmission, and composite endpoint. The reason may be patients with elevated HbA1c but without known diabetes likely have diabetes that was neither diagnosed nor treated, which causing worse prognosis without corresponding treatment.

And in the CAD patients with diabetes in our study, clinical depression and high HbA1c only led to more non-cardiac readmissions. Those patients with multiple major complications of diabetes and CAD are more likely to be treated with insulin and control the established risk factors such as hypertension and dyslipidemia, which are closely associated with MACE. This difference in treatment may partly explain the difference of outcomes. On the other hand, clinical depression is a psychological illness that can present with somatic symptoms. And HbA1c level has strong associations with dyslipidemia, hypercoagulability, and system inflammatory that may lead to multi-system diseases (26). So in addition to the cardiovascular system-related events, non-cardiac readmissions should also receive adequate attention and evaluation, which is essential to improve the quality of life and longevity of CAD patients.

Decreased exercise due to depression, sedentary lifestyle and poor medication adherence can all contribute to high glycation. But no relationship between depression and HbA1c was found. The insufficiency of sample, differences in grouping patterns and adjusted variables may influence the associations between depression and HbA1c. The relationship between them in CAD patients still needs further investigation.

Inflammatory activity may be a key mechanism of depression and HbA1c leading to poor prognosis in CAD patients. Whether controlling the systemic inflammatory state can improve the prognosis needs further study. Besides, increasing physical activity may be particularly beneficial for reducing depression and improving glycemic control at the same time (44). Moderate exercise may significantly improve the prognosis of CAD patients.

There are still several limitations in this study. At first, this prospective research was conducted in a single center from a southern central hospital, which can't represent patients in the entire China. Secondly, the small sample sizes of our study might lead to inaccurate results, especially in the diabetic and non-diabetic subgroups analysis. Thirdly, the prognostic information of most patients was obtained through telephone follow-up by our professional cardiologists, which might lead to inaccurate results of re-hospitalization and MACE. Finally, patients who experienced emergency percutaneous coronary intervention surgery were not included in this study, which might lead to an underestimation of the influence of high HbA1c and depression to poor prognosis.

## Conclusion

In conclusion, the presence of both depression and high HbA1c lead to a worse prognosis in CAD patients than one risk factor alone, no matter with or without the comorbidity of diabetes in these CAD patients. For patients with CAD and depression, lower HbA1c may be required.

## Data Availability Statement

The original contributions presented in the study are included in the article/Supplementary Materials, further inquiries can be directed to the corresponding authors.

## Author Contributions

HY surveyed all patients. WL, HM, YL, and YC collected and entered data into database. WL, QL, and HZ did statistical analyses. WL, HY, and QG wrote the paper. QG and HM were senior physicians principally responsible for the study. All authors read and approved the final manuscript.

## Funding

This research was supported by the grants from National Natural Science Foundation of China (No. 8160284), Natural Science Foundation of Guangdong Province (Nos. 2019A1515011224, 2021A1515011118, and 2021A1515011781), Start-up Funding of National Natural Science Foundation of China (Nos. 8207120182, 8207050582, 8217142362, and 8197091267), the National Key R&D Program of China (No. 2018YFC2001805), Guangdong Medical Science and Technology Research Fund, China (2019118152336191 and A2020017), Guangzhou Science and Technology Foundation and Application Foundation Research Project (Nos. 202102080368 and 202102080033), Traditional Chinese Medicine Bureau of Guangdong Province (20201008), Leading Medical Talents Project in Guangdong Province (Climbing Plan Special Fund) (No. KJ012019431), and High-level Hospital Construction Project of Guangdong Provincial People's Hospital (Nos. DFJH201811, DFJH201922, DFJH2020003, and DFJH2020029).

## Conflict of Interest

The authors declare that the research was conducted in the absence of any commercial or financial relationships that could be construed as a potential conflict of interest.

## Publisher's Note

All claims expressed in this article are solely those of the authors and do not necessarily represent those of their affiliated organizations, or those of the publisher, the editors and the reviewers. Any product that may be evaluated in this article, or claim that may be made by its manufacturer, is not guaranteed or endorsed by the publisher.

## Abbreviations

CAD: coronary artery disease
HbA1c: hemoglobin A1c
aHR: adjusted hazard ratio
CI: confidence interval
MACE: Major Adverse Cardiovascular Events
PHQ-9: Patient Health Questionnaire-9
CCR: creatinine clearance
HDLC: High-Density Lipoprotein Cholesterol.
