# Supplementary material for: Associations Among Depression, Hemoglobin A1c Level, and Prognosis in Patients With Coronary Artery Disease: A Prospective Study
Source: Front Psychiatry. 2022 Jun 16;13:815196. doi: 10.3389/fpsyt.2022.815196 (PMC9243435; doi:10.3389/fpsyt.2022.815196)
Supplement: Supplementary file 1 [file Data_Sheet_1.docx]

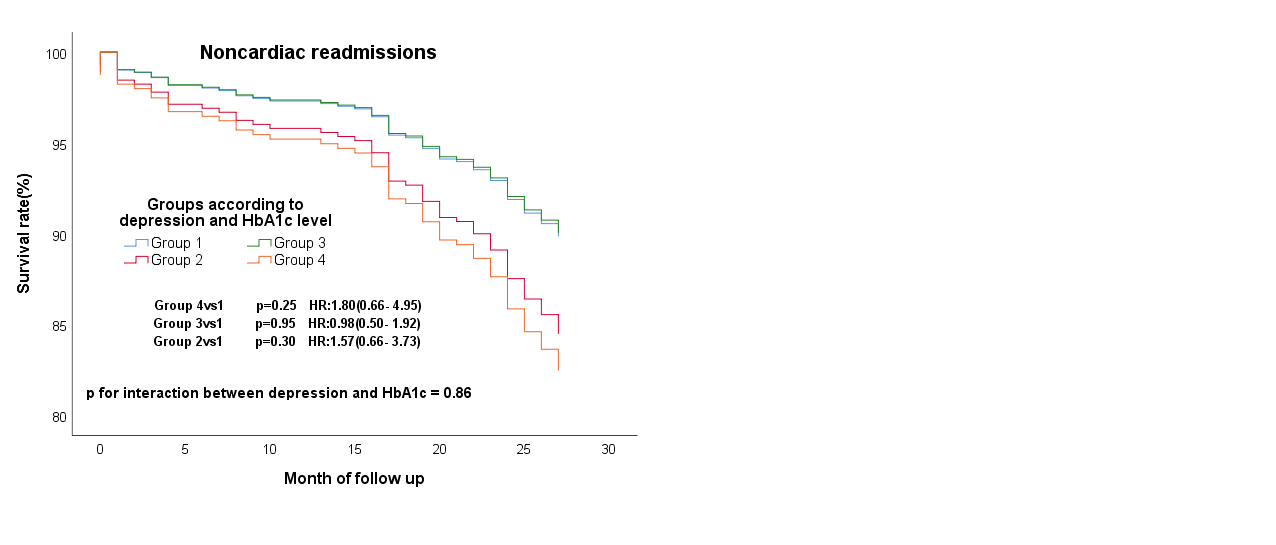


SUPPLEMENTARY FIGURE 1 | Cox regression curves for noncardiac readmissions by the four groups of all patients in the multi-variable adjusted model. (Group1: patients without clinical depression and low HbA1c; Group2: patients with clinical depression and low HbA1c; Group3: patients without clinical depression and high HbA1c; Group4: patients with clinical depression and high HbA1c). Noncardiac readmissions Model: Adjusted for sex, age, severity of coronary artery stenosis, diabetes, taking furosemide and CCR.


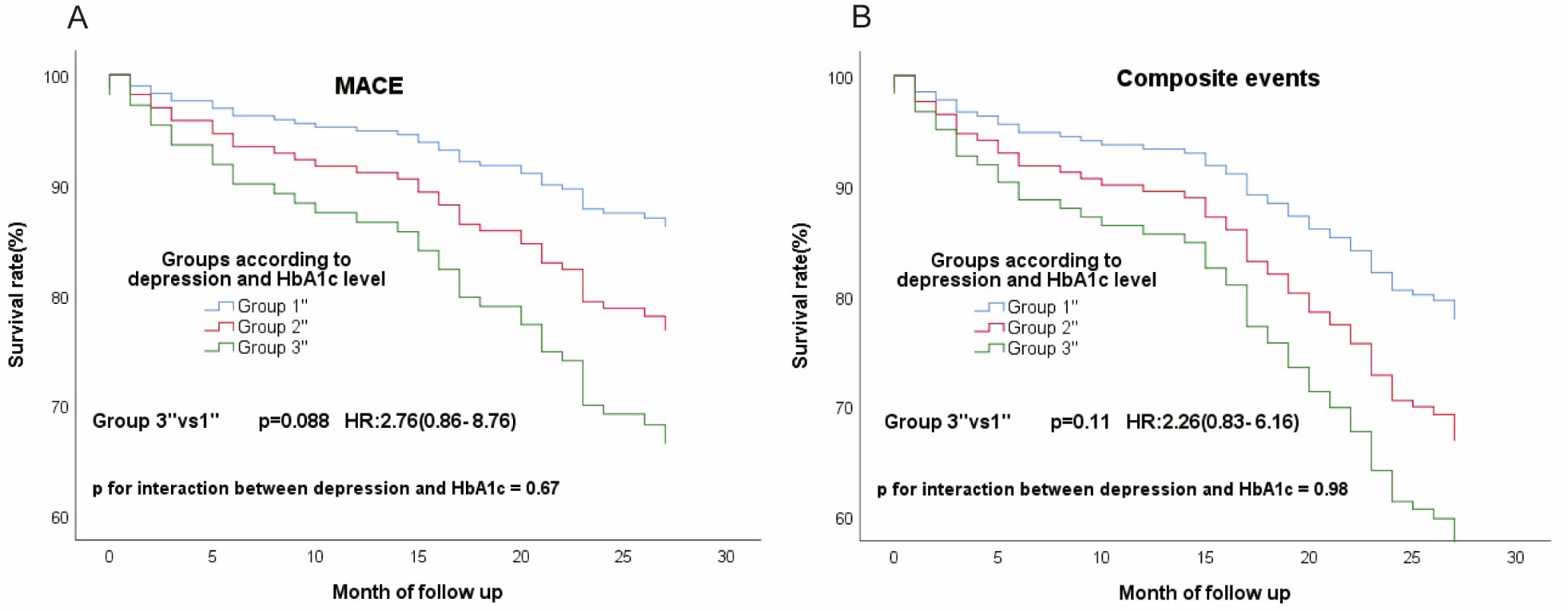


SUPPLEMENTARY FIGURE 2 | Cox regression curves for MACE (left) and composite endpoint (right) by the four groups of CAD patients with diabetes. (Group1’’ : patients without clinical depression and low HbA1c; Group2’’ : patients with clinical depression and low HbA1c, patients without clinical depression and high HbA1c; Group3’’ : patients with clinical depression and high HbA1c).
